# Ten best practices for effective phenological research

**DOI:** 10.1007/s00484-023-02502-7

**Published:** 2023-07-29

**Authors:** Richard B. Primack, Amanda S. Gallinat, Elizabeth R. Ellwood, Theresa M. Crimmins, Mark D. Schwartz, Michelle D. Staudinger, Abraham J. Miller-Rushing

**Affiliations:** 1 Department of Biology, Boston University, Boston, MA USA; 2 Department of Geography, University of Wisconsin-Milwaukee, Milwaukee, WI USA; 3 Department of Environmental Studies, Colby College, Waterville, ME USA; 4 iDigBio, Florida Museum of Natural History, University of Florida, Gainesville, FL USA; 5 Natural Museum of Los Angeles County, Los Angeles, CA USA; 6 USA National Phenology Network, School of Natural Resources and the Environment, University of Arizona, Tucson, AZ USA; 7 Department of the Interior, Northeast Climate Adaptation Science Center, US Geological Survey, Amherst, MA USA; 8 US National Park Service, Acadia National Park, Bar Harbor, ME USA

**Keywords:** Citizen science, Community science, Historical data, Mismatch, Phenology network, Remote sensing

## Abstract

The number and diversity of phenological studies has increased rapidly in recent years. Innovative experiments, field studies, citizen science projects, and analyses of newly available historical data are contributing insights that advance our understanding of ecological and evolutionary responses to the environment, particularly climate change. However, many phenological data sets have peculiarities that are not immediately obvious and can lead to mistakes in analyses and interpretation of results. This paper aims to help researchers, especially those new to the field of phenology, understand challenges and practices that are crucial for effective studies. For example, researchers may fail to account for sampling biases in phenological data, struggle to choose or design a volunteer data collection strategy that adequately fits their project’s needs, or combine data sets in inappropriate ways. We describe ten best practices for designing studies of plant and animal phenology, evaluating data quality, and analyzing data. Practices include accounting for common biases in data, using effective citizen or community science methods, and employing appropriate data when investigating phenological mismatches. We present these best practices to help researchers entering the field take full advantage of the wealth of available data and approaches to advance our understanding of phenology and its implications for ecology.

**Supplementary Information:**

The online version contains supplementary material available at 10.1007/s00484-023-02502-7.

## Introduction

The study of phenology, the seasonal timing of recurring life history events (Schwartz 2003), is increasingly used to investigate the effects of climate change and other environmental changes on ecosystems. Scientists from a range of disciplines are using phenology to study and predict the demographic consequences of species’ responses to climate change, the susceptibility of species to extreme weather events, and the effects of changes in growing season length on ecosystem processes such as water, nutrient, and carbon fluxes (Chmura et al. 2019; Poloczanska et al. 2013; Jin et al. 2017; Browning et al. 2021; Caparros-Santiago et al. 2021; Chuine and Régnière 2017; Iler et al. 2021a; Piao et al. 2019; Friedland et al. 2018).

New sources of phenological data are opening innovative avenues for research. The digitization of museum specimens, historical records, and field data allows scientists to examine phenological events over wider geographical areas and longer time scales than have previously been possible (Hedrick et al. 2020; Gwinn and Rinaldo 2009; Jarić et al. 2020). Rapidly expanding citizen science networks—such as eBird, iNaturalist, and Nature’s Notebook—are providing dense spatial and temporal coverage of certain phenological events, such as flowering and bird and fish migrations (Soroye et al. 2018; Taylor et al. 2019; La Sorte and Graham 2021; Dalton et al. 2022; Rosemartin et al. 2014). Long-term weather records, crucial to most phenological research, are becoming more accessible and can provide estimates of climate at fine geographical scales (Daly et al. 2002). Direct observations of phenology can be supplemented with camera images, sound recordings, and DNA samples, and matched to large-scale phenological data collected by satellites (Zeng et al. 2020; Yamasaki et al. 2017; Matsuhashi et al. 2019; Zimova et al. 2020a; Brown et al. 2016b; Buxton et al. 2016; Friedland et al. 2018).

As the study of phenology grows, researchers benefit from understanding best practices in designing phenology studies, working with data, and interpreting results. Many phenological data sets have peculiarities that are not immediately obvious and can lead to mistakes in analyses and interpretation of results. This challenge is exacerbated when researchers analyze data sets that lack good metadata (a common problem for historical data sets) or combine data from different sources such as citizen science, museum specimens, and remote sensing (Elmore et al. 2016; Atha et al. 2020). With the abundance of historical phenology data, new data becoming available, and new experiments and studies being designed, these challenges surface with increasing frequency in phenology research.

Here we present ten best practices that can help researchers overcome these challenges and advance the study of phenology through better planning, data collection, analyses, and interpretation. These practices are appropriate whether researchers use existing data, collect new data, or combine new and past data.

1. Ensure clear and consistent data collection protocols
2. Be aware of data quality and biases
3. Match data precision and duration to the question or application
4. Utilize citizen science data and programs to maximize research benefit
5. Avoid errors when combining or comparing disparate data sets
6. Account for long-term changes in the study species
7. Account for external factors that affect the study system
8. Use statistics and models appropriate for the data and questions
9. Ensure appropriate data are available when studying phenological mismatches
10. Base new phenological theories on more comprehensive evidence

## Materials and methods

This paper is the result of a working group (the authors) that synthesized decades of experience to help researchers new to the field avoid common problems when studying phenology. Working group members frequently encounter these issues when reviewing grant proposals and manuscripts, and even when reading published literature. The working group’s goal was to identify the most common challenges and suggest best practices that researchers could use to overcome these challenges. The working group identified the challenges and best practices based on (1) their own experiences reviewing proposals and manuscripts, (2) a review of the literature, and (3) input from other experts (see Acknowledgements). The resulting best practices are organized generally by activities that occur during planning and data collection (best practices 1–4) and activities that occur during data analysis and interpretation (best practices 5–10). Case studies that highlight practical use of these best practices are included as online supplementary information.

## Best practice 1: Ensure clear and consistent data collection protocols

### Challenges

**Fig. 1:**
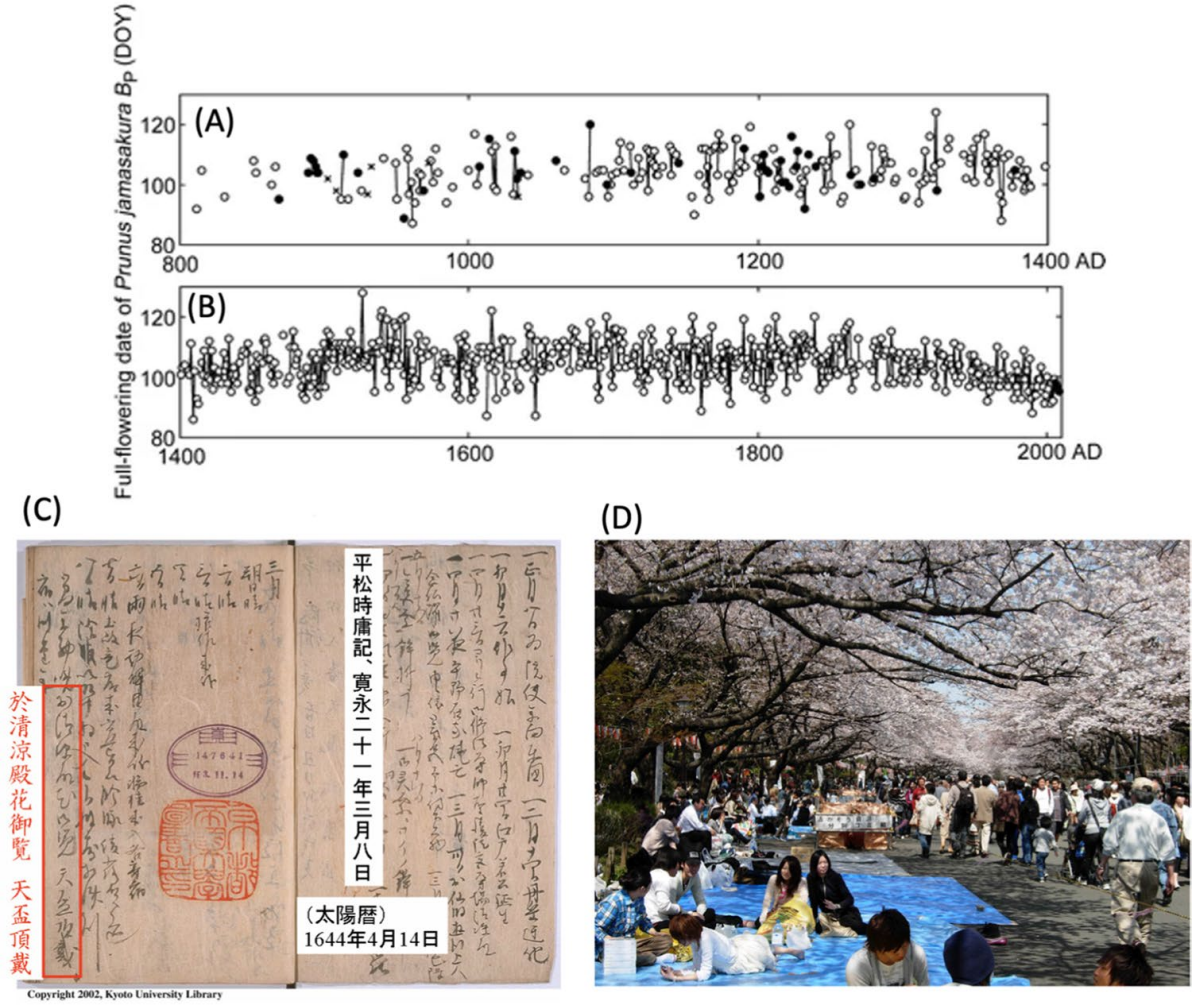
Interannual variation in the dates of the cherry blossom festival in Kyoto, Japan, shows earlier flowering times in the past 50 years; data were acquired from historical documents and recent records. Dates of full flowering of the mountain cherry (*Prunus jamasakura*) are shown for **A** the period from AD 801 to 1400 and **B** 1400 to 2008. Flowering dates are affected by both global climate change and urbanization. Data gaps exist for particularly old records, many of which were lost during natural disasters and fires. DOY refers to day-of-the-year (i.e., Jan. 1 = 1). Language and calendars changed during this record, requiring researchers to translate old records to modern Japanese language and the Gregorian calendar. **C** Historical record documenting cherry flowering. **D** People celebrating the cherry blossom festival in Tokyo. Figure from (Aono and Saito 2010; Aono and Kazui 2008) (images courtesy of Aono (**A**–**C**) and Hiroyoshi Higuchi (**D**))

Researchers often do not have control over protocols used (previously) to collect the data they analyze (e.g., when analyzing historical or citizen science data or during meta-analyses). Sampling protocols and effort might change over the data collection period, or metadata may lack key information. For example, exceptionally long-term records of cherry blossom festivals in Kyoto, Japan, suffer from changes in protocols and gaps in data (Fig. 1) (Arakawa 1956; Aono and Saito 2010). And long-term marine studies frequently experience changes in survey gear that affect species catchability (Staudinger et al. 2019). Variation in sampling timing (e.g., dates of collection), intensity (e.g., days per week or number of sites), location, or significant gaps in long-term time series can affect detection of extreme events (e.g., unusual dates of first or last flowering or bird arrivals) (Leopold and Jones 1947; Bradley et al. 1999) and may yield results that reflect changes in sampling rather than phenology (Stegman et al. 2017; Schwartz et al. 2013; de Keyzer et al. 2017; Dalton et al. 2022). These challenges can extend to contemporary data as well. For example, observers of bird phenology may not record whether they use visual or audible cues, how many hours they observe, or how large of an area they survey.

### Best practices

When collecting data, it is important to follow clear and consistent protocols that are thoroughly documented, readily available, and updated as needed (Nordt et al. 2021; Denny et al. 2014). Ideally, documentation includes illustrations or photos to aid identification of species or phenophases. When possible, researchers should cross-reference their protocols to international phenology standards, such as the BBCH system for plants (Meier 2003; Meier et al. 2009), which can aid comparisons across data sets.

In the absence of good metadata, it may be possible to contact people who have worked with the data previously or use clues from the data or field notes to reconstruct methods. Researchers can also use statistical techniques, such as correction factors or hierarchical survival models, to account for changes in methods (Miller et al. 2021; Elmendorf et al. 2019; Moritz et al. 2008; Dalton et al. 2022). However, researchers should exercise caution when interpreting results derived from poorly documented data (Online Resource 1).

## Best practice 2: Be aware of data quality and biases

### Challenges

Phenological observations can suffer from imperfect detection and misidentification of species and phenophases (McDonough MacKenzie et al. 2017), introducing error into data sets. Detection and identification challenges can occur whether observations are made by volunteers or professionals, or are extracted from historical records. Fuccillo et al. (2015) found that volunteer observers identified phenophases correctly more than 90% of the time, though accuracy dropped during periods of transition (e.g., transition from closed to open flowers). Highly mobile, cryptic, or aquatic species can introduce uncertainty due to imperfect detection and the risk of false non-detections (Ramp et al. 2015; Staudinger et al. 2019; MacKenzie and Royle 2005).

Observer preferences, which can reflect study goals or individual habits, can bias data in a variety of ways (Online Resource 2). For example, observers may preferentially observe plants or animals that are more accessible, biasing observations towards those closer to buildings, shorelines, roads, or cities, or toward individuals that are more visible or appear healthier than nearby counterparts (Daru et al. 2018; Hijmans et al. 2000; Cohen et al. 2018). Observers may also choose to track species that are easier to identify or are more eye-catching (Hortal et al. 2007). Observers may favor or avoid rare species (Daru et al. 2018; Callaghan et al. 2019). Or observers may favor some phenophases over others (e.g., insect flight times rather than larval development) (Rosemartin et al. 2018; Crimmins et al. 2022). In many cases, logistical constraints may dictate preference for observing certain life stages; for example, phenology of immature seabirds, migratory whales, and larval stages of many fish and invertebrate species are difficult to observe (Staudinger et al. 2019; Pendleton et al. 2022). Finally, temporal bias can arise if observers concentrate effort on particular days of the week, seasons, during favorable weather, or during organized events like World Migratory Bird Day or bioblitzes (Daru et al. 2018; Courter et al. 2013; Crimmins et al. 2021).

As with all data collection, errors can arise when observers record data or when people or software transcribe data from paper to digital formats (e.g., 6/7 could mean June 7 or July 6). Errors in recording data may occur more frequently early in growing seasons, when many observers are first learning field methods (Crimmins et al. 2017), or in the first year for particular observers.

In addition, phenology observations collected from controlled experiments may not reflect “real world” phenology (Wolkovich et al. 2012). Bias in experimental results can be caused by methods of warming (e.g., open-top greenhouses, soil warming, warming chambers) that fail to replicate field warming, by methods that alter humidity and soil moisture in unexpected ways, or by methods that warm only parts of plants or study plants at different life stages (Wolkovich et al. 2012; Primack et al. 2015; Berend et al. 2019). Similarly, manipulations of precipitation (e.g., rain catchment systems) can influence sunlight or wind, and manipulations of snowmelt date (e.g., dust, snow removal, or tarps) can influence nutrients, moisture, and carbon dioxide emissions (Beier et al. 2012; Rixen et al. 2022).

### Best practices

Data users should carefully consider sources of phenological data, potential biases, and the impact of these factors on results (Online Resource 2). The use of vouchers—such as photographs, specimens, genetic samples, or environmental DNA (eDNA)—can improve detections and species identification (Ogden 2022; Zimova et al. 2020a). The use of algorithms or custom applications can further support species identification where large data sets of photographs are available, such as in the case of digitized museum specimens or photo-based citizen science programs, like iNaturalist or iSpot (Puchałka et al. 2022). Including observer identity and expertise level in metadata can allow data users to track and account for some types of observer bias. Setting minimum limits on temporal or geographical representation (e.g., a minimum number of observations from each time period or area) and averaging across observations can reduce the likelihood of single observations unduly influencing results (Puchałka et al. 2022). Statistical estimators (e.g., Weibull estimators) can help account for some observer biases, particularly for non-systematically collected data, although they may not account for environmental heterogeneity (Pearse et al. 2017; Iler et al. 2021b). Repeated measures designs can also help control for observers, which may be important in some data sets.

Combining experimental and observational approaches to studying phenology can help avoid biases caused by experimental or observational methods alone (McDonough MacKenzie et al. 2020). Areas where experiments and observations disagree can reveal potential biases or confounding factors that deserve further investigation or should be accounted for in analyses (Rixen et al. 2022) (see also Best Practice 5 for more information about combining or comparing disparate data sets).

## Best practice 3: Match data precision and duration to the question or application

### Challenges

Over the course of a year, organisms progress through major phenophases such as growth or reproductive states as well as finer-scale stages within those major phases (Meier 2003). For example, leaf development involves expansion of leaf bud scales, emergence of young leaves, and numerous stages of growth. Marine fish pass through phenophases such as immature, developing, active spawning (ripe and running), and atresia (reproductive cessation). Researchers might record the timing of finer-scale phenophases in the hopes that increased detail will provide more useful data. However, for many research applications, coarser-scale phenophases are sufficient and can save observers and researchers time and effort (Ellwood et al. 2019; Pearson 2019; Nordt et al. 2021).

Additionally, “noise” or variability in data can mask phenological responses to climate change. This variability can be caused by a variety of factors, including differences among observers (Best Practice 2), microsite variation, the duration of particular phenophases, and extreme weather and other environmental events (Casson et al. 2019; Feiner et al. 2022). When using climate data to simulate or forecast phenology, linear models can miss nonlinear responses to climate that can occur at cold or warm extremes within species tolerances, such as can occur when species fail to meet winter chilling requirements (Ibáñez et al. 2010; Ettinger et al. 2020). Thus, drawing conclusions from linear models or short-term or small-scale studies can risk flawed interpretations of findings (Bolmgren et al. 2012).

### Best practices

We find that most studies benefit from long-term monitoring of major phenophases. Long-term or large-scale data sets generally provide more accurate estimates of phenological sensitivity to environmental drivers (Primack et al. 2009; Gallinat et al. 2018; Bolmgren et al. 2012). However, some research questions require finer-scale phenology observations or can be addressed by short-term studies that contrast warm and cool or wet and dry years, sites along elevational gradients, or sites with different land uses (McDonough MacKenzie et al. 2019; Berend et al. 2019; Jia et al. 2021). Researchers also have access to high quality climate data and phenology data for many species, which can help when developing appropriate quantitative models to study phenology (Ettinger et al. 2020). Researchers may consider power analyses and simulations to help identify the appropriate level of detail and duration for their research questions and avoid erroneous conclusions (Meyer et al. 2010; Bolmgren et al. 2012).

## Best practice 4: Utilize citizen science data and programs to maximize research benefit

### Challenges

Programs focused on citizen science, sometimes called community science, public participation in science, or other terms (Eitzel et al. 2017; Cooper et al. 2021), have dramatically increased the geographic and taxonomic breadth of phenological observations available for scientific research. These programs include qualitative narratives (ISeeChange), incidental reports (iNaturalist, iSpotNature), and repeated measurements of individual plants, plots, or waterbodies (Nature’s Notebook, Budburst). Researchers also sometimes develop their own citizen science projects or campaigns and data collection platforms (Young et al. 2021). The variety of methods, levels of observer expertise, and other factors can present challenges when selecting or designing citizen science programs and analyzing data (see also Best Practices 1 and 2). With the range of options for engaging with citizen science, it can be difficult for researchers to identify the best approach to meet their needs.

### Best practices

Researchers can reach out to existing phenology networks and citizen science associations to help identify citizen science approaches most appropriate for their study goals (Online Resource 3) (Storksdieck et al. 2016). Researchers implementing new citizen science and other monitoring programs can minimize common sources of errors (e.g., misidentification or miscounting) by training volunteers, providing clear reference materials, having experts validate observations, encouraging replication of observations, and communicating results or otherwise engaging with volunteers (Kosmala et al. 2016; Robinson et al. 2021). Researchers should carefully plan for the resources necessary when designing their own citizen science projects; projects frequently require more initial resources than expected (McKinley et al. 2017). Adapting existing citizen science programs—such as iNaturalist, eBird, or iSpot—or collaborating with phenology networks can be efficient approaches for researchers to incorporate citizen science into their own research, enhance those programs, and increase public engagement in ecological studies of climate impacts (Online Resource 3).

## Best practice 5: Avoid errors when combining or comparing disparate data sets

### Challenges

As phenology data sets become more accessible, researchers frequently combine or compare data from different sources (Peng et al. 2017; Gill et al. 2015; Kharouba et al. 2018; Keogan et al. 2022). Linking continental-scale phenology with the phenology of ecological communities or single species is a major challenge for the emerging field of macrophenology (Gallinat et al. 2021). However, problems can arise when researchers do not carefully account for the features or biases of diverse data at different scales. Such varied data are not easy to integrate—satellite data typically integrate phenology of many species within pixels, while ground-based observations generally include small numbers of individuals at specific sites.

Data sources can also record different phenophases—such as the beginning, peak, or end of flowering or breeding—the timing of which may be correlated but could be influenced by different factors (CaraDonna et al. 2014; Iler et al. 2021b; Keogan et al. 2022). For instance, first and last dates can be influenced by changes in population size, age, body size, or sex (Peer and Miller 2014; Cohen et al. 2018; Dalton et al. 2022), while peak dates may be more strongly related to environmental conditions (Miller-Rushing et al. 2008b). Even data sets using similar methods can be influenced by small differences in phenophase definitions employed by different researchers and observation networks (Schwartz et al. 2006).

### Best practices

**Fig. 2:**
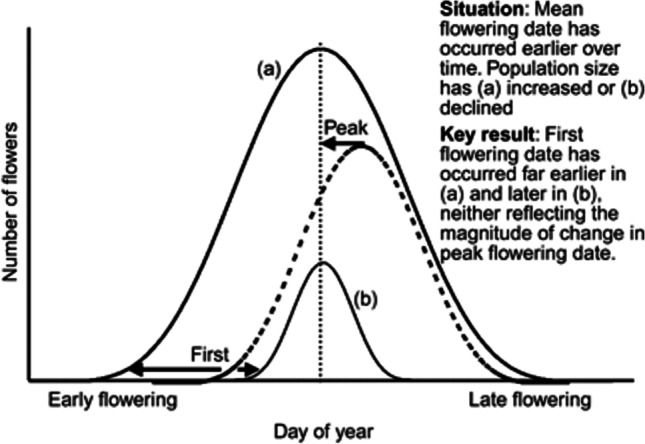
The theoretical effect of changes in population size on changes in first dates of phenological events, assuming constant sample effort. Dashed curve shows the distribution of phenological event dates for a population in a past year. The solid curves show two scenarios in which the mean date of the population occurs earlier now than it did in the past: the population size has either (a) increased or (b) declined. Arrows highlight changes in peak and first dates over time. Increases or decreases in sampling effort could similarly affect observed dates. Figure is not drawn to scale and is conceptual only. Miller-Rushing et al. (2008b) found empirical evidence for this phenomenon in bird populations in eastern Massachusetts, USA

Researchers synthesizing data types (e.g., historical observations, museum specimens, remote sensing, citizen science) should become familiar with the specifics of the data and metadata (Stucky et al. 2018). It is important to reconcile any differences in the terminology that different data sets use and the stages of phenology they measure (Stucky et al. 2018). In general, it is helpful to use data that document peaks of phenophases, rather than first or last phenological events, which can be influenced by several other factors (Fig. 2). Investigators can also seek out data sets where sample sizes are available to estimate confidence intervals in phenology dates, durations, and trends (Puchałka et al. 2022; Li et al. 2021).

Emerging techniques can help researchers integrate data across spatio-temporal scales and multiple species or ecological communities (Online Resource 4). For example, cameras or direct observations can capture the phenology of individuals or small populations of plants or animals; phenocams and drones can capture phenology at landscape scales; and satellite imagery can capture phenology at even larger landscape, continental, or global scales. Combining data across these scales is not trivial (Online Resource 4), but the ability to study these scales simultaneously opens new opportunities for research (Gallinat et al. 2021).

## Best practice 6: Account for long-term changes in the study species

### Challenges

Changing population size can affect observations of first and last phenological events in a year, such as animal migrations and plant flowering (Fig. 2) (Tryjanowski and Sparks 2001; Tryjanowski et al. 2005; Miller-Rushing et al. 2008a; Koleček et al. 2020; Dalton et al. 2022). Because of variation in phenology within populations, first events tend to occur earlier and last events tend to occur later as population sizes increase, regardless of changes in the average timing of the events (de Keyzer et al. 2017; Tillotson and Quinn 2018). The reverse happens when populations decline (Miller-Rushing et al. 2008b). Detectability of first and last events also tends to increase as species become more abundant (Koleček et al. 2020) or due to within population demographic (e.g., sex ratios) changes (Peer and Miller 2014). This issue is generally of less concern when monitoring tagged individuals (thus avoiding the influence of changes in population size), but can be a problem, for example, if tagged plants increase in size, increasing the number of flowers and advancing the time that the first flowers open each year.

Over long time periods, genetic changes can also influence the phenology of species, making it difficult or impossible to disentangle the contributions of genetic and plastic responses to environmental cues when explaining long-term changes in phenology. This is particularly true for species with short generation times, such as plankton, insects, and annual plants (Colautti and Barrett 2013; Manhard et al. 2017; Lustenhouwer et al. 2018). Climate-driven behavioral responses in long-lived animals, such as shifts in diurnal or seasonal behaviors to utilize different microclimates or prey resources, can further complicate detectability and interpretation of the mechanisms driving changes in phenology (Teitelbaum et al. 2021; Beever et al. 2017; Pendleton et al. 2022).

### Best practices

Researchers frequently recommend the use of population means or entire distributions of phenological events to assess changes in phenology over time (Miller-Rushing et al. 2008a; Moussus et al. 2010; CaraDonna et al. 2014). However, many long-term phenology data sets contain only first observations, such as when the first bird of a species arrives in the spring (Inouye et al. 2019). Researchers analyzing changes in first events must consider the influence of changes in abundance, demography, and genetic structure, which they can do, for example, by including population size, sex, or body size as a covariate in statistical analyses. This is particularly important for studying invasive species, rare species, and managed species that are recovering, many of which are rapidly increasing or declining in abundance. Sometimes it may not be obvious whether changes in abundance are influencing changes in phenology or vice versa, so researchers must take care when identifying the directionality of cause and effect (Willis et al. 2008, 2010; Cleland et al. 2012; Colautti and Barrett 2013).

## Best practice 7: Account for external factors that affect the study system

### Challenges

A number of external factors, aside from the effects of climate change, can influence plant and animal phenologies. For example, changes in land use, land cover, and habitat connectivity—such as those caused by urbanization, dams, and restoration—can directly affect local temperatures and phenology and can obscure, mitigate, or compound the effects of climate change on phenology (Meng et al. 2020). Such changes may also affect phenology indirectly through changes in population size or altering access to and the quality of habitats (Miller-Rushing et al. 2008a, b). For example, urbanizing areas might warm more rapidly than surrounding areas, creating islands of relatively early phenology (Zhang et al. 2004; Neil and Wu 2006; Møller et al. 2015; Chick et al. 2019). Such islands of early phenology could complicate assessments of landscape phenology, phenology-related ecosystem functions, and the potential for phenological mismatches.

Barriers to migration could reduce access to microclimates and critical resources such as spawning or breeding habitat (Mattocks et al. 2017). Unexpected changes in phenology might also occur if winter chilling requirements are not met during a mild winter (Wilson et al. 2016; Pierson et al. 2013; Zimova et al. 2020b), if microclimates create refugia from warming temperatures (Li et al. 2019; Meng et al. 2020), or if food availability changes in time or location. Such changes in climate, microclimate, or food availability can create difficult-to-explain variation in studies that do not consider winter chilling, assume relatively uniform temperatures across localities, or fail to consider influences of food availability or other factors on animal phenology. In other cases, areas that were formerly open grazing or woodlands have become closed-canopy forests, cooling understories, and delaying phenology (Li et al. 2015). These delays in phenology would be difficult to explain without understanding the landscape history.

Changes in species composition, which occur through succession and restoration, can confound interpretation of phenology. For example, when species are difficult to distinguish from each other visually or through remote sensing, changes in phenology may simply reflect changes in the relative abundance of species with differing phenologies (Helman 2018). These problems can arise even in areas where land use and cover are not changing, such as areas where nonnative invasive plants are increasing in abundance or when closely related species co-occur in unknown ratios (e.g., species of river herring: alewife *Alosa pseudoharengus* and blueback *Alosa aestivalis*). Many invasive plant species differ in phenology from native species, leafing out earlier in the spring or senescing later in the autumn (Fridley 2012; Reeb et al. 2020), which can challenge efforts to identify factors driving changes in vegetation phenology observed by remote sensing.

### Best practices

In many cases, researchers can take advantage of external factors to address questions or species that might otherwise be difficult to address. For example, warming in urban areas, which is accelerated by the urban heat island effect, may allow researchers to anticipate how the phenology of more slowly warming surrounding rural areas may be affected by future climate change. Narrow migration points, such as fish ladders on dams and wildlife overpasses on highways, can provide opportunities to monitor mobile species that are otherwise cryptic and difficult to monitor.

## Best practice 8: Use statistics and models appropriate for the data and questions

### Challenges

Researchers commonly use linear regression models and growing degree-day models to examine historical changes in phenology over time and in relation to climate variables such as temperature (Roberts 2012). The results of linear regression models (e.g., reported as days/decade or days/°C) are easy to interpret, compare, and communicate. However, linear models have limitations. For instance, as species reach physiological limits under new environmental conditions, phenological responses may be nonlinear (Iler et al. 2013; Fu et al. 2015; Ibáñez et al. 2010).

Phenological studies often include data from many species, and analyses of these data sometimes treat species as independent points. However, closely related terrestrial plant species often share similar phenology and similar phenological responses to climate change (Willis et al. 2008; Davies et al. 2013; Panchen et al. 2014). Analyses that treat species independently and fail to account for phylogenetic relationships may unintentionally inflate sample sizes and confidence in their results. Conversely, in aquatic ecosystems, phenological responses and drivers may vary widely among closely related species or even among populations of the same species (Staudinger et al. 2019; Legett et al. 2021; Dalton et al. 2022; Walsh et al. 2015). Furthermore, phylogenetic signals in response to climate change may be absent in some plant communities (CaraDonna and Inouye 2015). The role of phylogeny in phenological responses to climate change appears to vary considerably across taxa and communities.

### Best practices

Multivariate models can statistically control for confounding factors such as changes in population size and sampling effort (Dalton et al. 2022), varying spatial scales of data sets (Zimova et al. 2020c) and spatial autocorrelation, accounting for phylogenetic relationships, and including broad-scale processes such as oceanographic and atmospheric circulation (Staudinger et al. 2019; Thaxton et al. 2020). Circular statistics can help analyze changes in phenology in areas without clear seasonal transitions, where plants may be continuously active, such as many tropical or subtropical areas (Rafferty et al. 2020). Mechanistic models, including growing day-degree models, can also highlight factors contributing phenological shifts and improve out-of-sample predictions (Chuine et al. 2003; Ettinger et al. 2020). Ecological forecasting techniques can incorporate statistical and mechanistic approaches to test the accuracy of predictions as new observations become available (Taylor and White 2020). Ultimately, the appropriate complexities and structures of models should be carefully selected according to study goals and the need to explain variation, provide accurate predictions, and be straightforward to communicate (Brown et al. 2016a; Tredennick et al. 2021).

## Best practice 9: Ensure appropriate data are available when studying phenological mismatches

### Challenges

In many cases, ecologically interacting species have similar phenological responses to climate change. However, phenological mismatches can occur when the phenologies of interacting species shift in different directions or magnitudes, disrupting ecological interactions. Such disruptions could have significant consequences for population dynamics, community structure, or ecosystem functioning. For instance, pied flycatchers (*Ficedula hypoleuca*) have experienced population declines of up to 90% as a result of temperature-driven shifts in the timing of their insect prey (Both et al. 2006). In some cases, asynchrony can benefit the fitness and survival of one or more of the species involved, such as in plant–herbivore or predator–prey interactions.

Most studies of mismatch cite differences in how interacting species or taxonomic groups’ phenologies are responding to changing climate conditions. These syntheses often suggest weaker climate sensitivity in secondary consumers compared to other trophic levels (Thackeray et al. 2016) and suggest mismatches are more likely in antagonistic interactions than mutualisms (at least in terrestrial ecosystems). This is because mutualists sometimes have co-adapted phenological triggers (Renner and Zohner 2018). However, few studies have demonstrated links between asynchrony and changing population demographics and fitness (Miller-Rushing et al. 2010; Visser and Gienapp 2019; Johansson et al. 2015; Kharouba and Wolkovich 2020).

### Best practices

Two recent studies propose rigorous tests for diagnosing whether mismatches are occurring, including demonstrating that asynchrony is driven by environmental change and that asynchrony impacts the fitness and survival of the populations involved (Kharouba and Wolkovich 2020; Samplonius et al. 2021). Demonstrating causal links between environmental changes, shifts in phenology, and changing population dynamics requires extensive data that are not readily available for most species’ interactions. However, these data requirements can help researchers focus on systems where such data do exist or where appropriate experiments are possible. Despite the importance of advancing connections between phenological mismatch and population responses, researchers should not have so strict a definition of mismatch that it is impossible to explore when important asynchronies occur—overly narrow definitions may hinder progress in the field.

## Best practice 10: Base new phenological theories on more comprehensive evidence

### Challenges

The vast majority of phenological research is based in temperate regions of North America, Europe, Australia, and increasingly East Asia because of the concentration of research capacity, funding, and long-term data in these regions (Wolkovich et al. 2014; Cohen et al. 2018). Phenology is understudied in other regions and systems, including marine, tropical, and arid ecosystems. In temperate terrestrial ecosystems, seasonal differences in temperature generally lead to distinct phenophases with noticeable beginnings—e.g., spring flowering and leaf out, insect emergence, and bird migration—that are generally advancing as temperatures warm. Methods and findings from these regions are not necessarily transferable to other regions or to marine systems, where organisms may exhibit different annual patterns, respond to different environmental cues, or exhibit seasonal lag-effects (Staudinger et al. 2019; Dalton et al. 2022; Henderson et al. 2017). In the tropics, for example, plant leafing and flowering cycles can occur more than once in a year or once every several years (Sakai and Kitajima 2019). The lack of phenological research in many regions makes it difficult to predict future changes in phenology and to understand the impacts changes in phenology are having on local ecology and on Earth systems (Cook et al. 2012; Staudinger et al. 2019; Gallinat et al. 2021).

### Best practices

New phenology projects and networks should be initiated in less studied regions and ecosystems. Even in well-studied temperate systems, researchers should challenge assumptions about factors that drive phenology, as some existing generalities are biased towards certain taxonomic groups, habitats, regions, and environmental factors (Brown et al. 2016a; Cohen et al. 2018). For instance, Zani et al. (2020), using a combination of data from experiments and the Pan European Phenology Project, proposed that an annual limit on carbon sequestration of tree leaves can limit growing season length; however, this new theory has been challenged using other data sets (Lu and Keenan 2022).

## Conclusions

Best practices in phenological research will continue to evolve with new research (Pearse et al. 2017; Iler et al. 2021b). New technologies and increased data availability—including from new and underused sources—will undoubtedly lead to the development of new data and analytical tools. For example, machine learning technology holds great potential for evaluating digitized museum specimens and images collected from camera monitoring (Zimova et al. 2020a); eDNA and other molecular techniques can capture occurrences of migratory and cryptic species (Ogden 2022; Yamasaki et al. 2017); and remotely sensed images can describe global phenological patterns (Xin et al. 2020; Pearson et al. 2020; Friedland et al. 2018). Work in regions where phenology is understudied (e.g., tropical, arid, and marine ecosystems) will also provide new insights. However, analyses of these data sets must be accompanied by good research practices such as those detailed here. These best practices are not always obvious, but when followed can significantly improve the conclusions and impact of phenological research.

## Supplementary Information

Below is the link to the electronic supplementary material.

Supplementary file1 (PDF 10595 KB)
