# Supplementary material for: Ten best practices for effective phenological research
Source: Int J Biometeorol. 2023 Jul 29;67(10):1509–22. doi: 10.1007/s00484-023-02502-7 (PMC10457241; doi:10.1007/s00484-023-02502-7)
Supplement: Supplementary file 1 — Supplementary file1 (PDF 10595 KB) [file 484_2023_2502_MOESM1_ESM.pdf]

Supplementary Information

Ten best practices for effective phenological research

Richard B. Primack[1,*], Amanda S. Gallinat[2,3], Elizabeth R. Ellwood[4,5], Theresa M. Crimmins[6], Mark D. Schwartz[2], Michelle D. Staudinger[7], Abraham J. Miller-Rushing[8]

[1]Department of Biology, Boston University, Boston, Massachusetts, USA
[2]Department of Geography, University of Wisconsin-Milwaukee, Milwaukee, Wisconsin, USA
[3]Department of Environmental Studies, Colby College, Waterville, Maine, USA
[4]iDigBio, Florida Museum of Natural History, University of Florida, Gainesville, Florida, USA
[5]Natural Museum of Los Angeles County, Los Angeles, California, USA
[6]USA National Phenology Network, School of Natural Resources and the Environment, University of Arizona, Tucson, Arizona, USA
[7]US Geological Survey, Department of the Interior, Northeast Climate Adaptation Science Center, Amherst, Massachusetts, USA
[8]US National Park Service, Acadia National Park, Bar Harbor, Maine, USA

*Corresponding author: Primack, R.B. (primack@bu.edu)

This Supplementary Information file contains Online Resources 1-5, each of which describes a case study illustrating best practices provided in the main text:

Online Resource 1 – *Case study – Using data sets with poorly described methods to understand phenological change*

Online Resource 2 – *Case study – Geographical biases in data in Denmark*

Online Resource 3 – *Case study – Phenology networks and resources for citizen science*

Online Resource 4 – *Case study – Assessing phenology across spatial scales*

Online Resource 5 – *Case study – Tracking seasonal migrations of anadromous fishes*

Online Resource 1

Case study – Using data sets with poorly described methods to understand phenological change

Henry David Thoreau, the environmental philosopher, made detailed observations of flowering, tree leaf out, and bird arrival times for hundreds of species from 1851 to 1858 around Concord, Massachusetts, USA (Primack and Miller-Rushing 2012). Other naturalists and researchers continued making these observations over the past 160 years, providing an opportunity to investigate the effects of climate change across trophic levels (Fig. S1) (Ellwood et al. 2021).

Unfortunately, methodologies used by different observers are poorly described. Thoreau documented some of his methods, such as flowering dates, in his detailed journals. Other methods, such as sampling intensity, can be inferred from the data themselves, but other aspects of his methods and those of subsequent observers are more difficult to determine. Did they use bird calls or visual sightings to detect first birds arriving each spring? What definitions did they use for particular phenophases, such as leaf out?

As another challenge, the combined data sets cover many decades but are non-continuous; they are missing data for some species in some years. Researchers had to normalize the data to account for variation among species (Ellwood et al. 2010).

Despite challenges with data quality, some striking patterns emerged. Spring phenologies of trees and wildflowers are responding more strongly to a warming climate than the arrival times of birds—so much so that these differences are readily apparent despite inconsistencies with methodology (Ellwood et al. 2010). The difference between canopy and understory phenology, however, suggests the possibility for important ecological mismatches and less time for wildflowers to take advantage of understory light in the spring (Heberling et al. 2019). The research team is using field experiments to test this possibility.

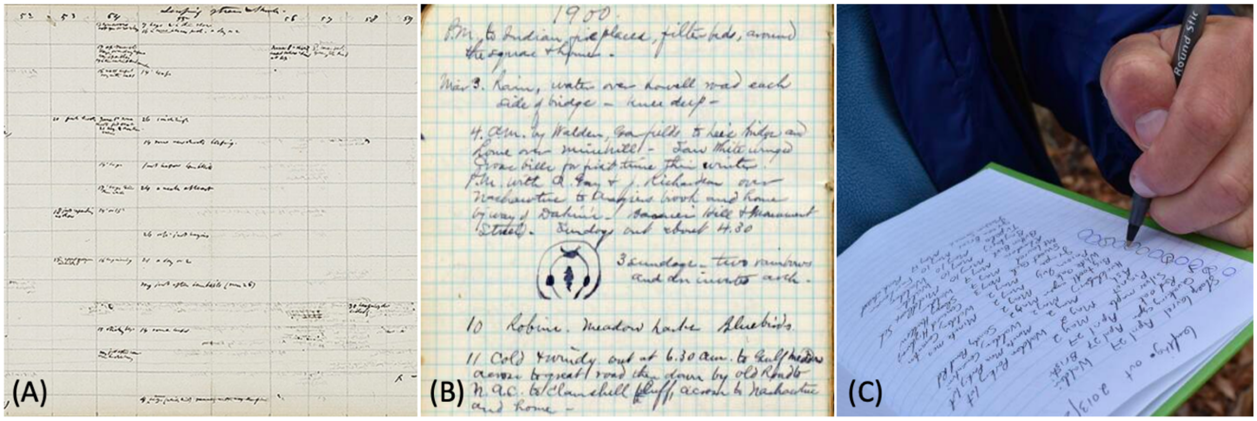

Fig. S1 Images of phenology journals of (A) Henry David Thoreau from the 1850s, (B) Alfred Hosmer from around 1900, and (C) Richard Primack in recent decades documenting flowering phenology in Concord, Massachusetts, USA. Images courtesy of Concord Museum (A), Concord Free Public Library (B), and WGBH (C)

Online Resource 2

Case study – Geographical biases in data in Denmark

Iwanycki Alstrand *et al*. (2022) recently used data from three different sources to study flowering phenology in Denmark. The data sets included 110 herbarium specimens spanning 145 years; 110 observations of first flowering data collected from the Exploring Denmark citizen science program in a single year; and 403 photographs of plants in flower taken over one year and uploaded to the iNaturalist citizen science program. Each data set included flowering dates of three common plant species: *Allium ursinum* (wild garlic), *Aesculus hippocastanum* (horse chestnut), and *Sambucus nigra* (black elderberry).

All three data sets showed strong geographical biases (Fig. S2), with collections concentrated in cities, with lower representation in the interior of the mainland. iNaturalist provided the most extensive geographic coverage, mainly due to the larger number of observations, a strength of many opportunistic citizen science programs (Soroye et al. 2018; Henckel et al. 2020). The herbarium specimens and iNaturalist photographs had comparable flowering dates as they mainly reflect peak flowering dates, suggesting they could be combined for further analysis. In contrast, Exploring Denmark citizen science recorded first flowering dates, which occurred much earlier (3-7 weeks earlier), and cannot reliably be combined with the other data.

Flowering dates were related to the average temperature in March, April, and May in the year and location of the observation (Fig. S2). Herbarium data reflected the strongest effect of temperature on flowering in Denmark, possibly because it included interannual variation in temperature. In contrast, Exploring Denmark citizen science observations of first flowering showed no effects of temperature, likely due to having only one year of data and a smaller sample size, and possibly because of other factors that might influence first flowering dates (Miller-Rushing et al. 2008).

In this study (Iwanycki Ahlstrand et al. 2022), combining herbarium and iNaturalist data provided the most effective method for detecting climatic effects on phenology. The combined data set included both spatial and temporal variation and showed that plants flower approximately 5 days earlier for each 1°C warming (Fig. 2). Phenology observations from citizen science programs like Explore Denmark and iNaturalist will increase in value for climate change research as additional years become available, particularly if people are encouraged to make observations from parts of the country that are currently under-represented in the data sets.

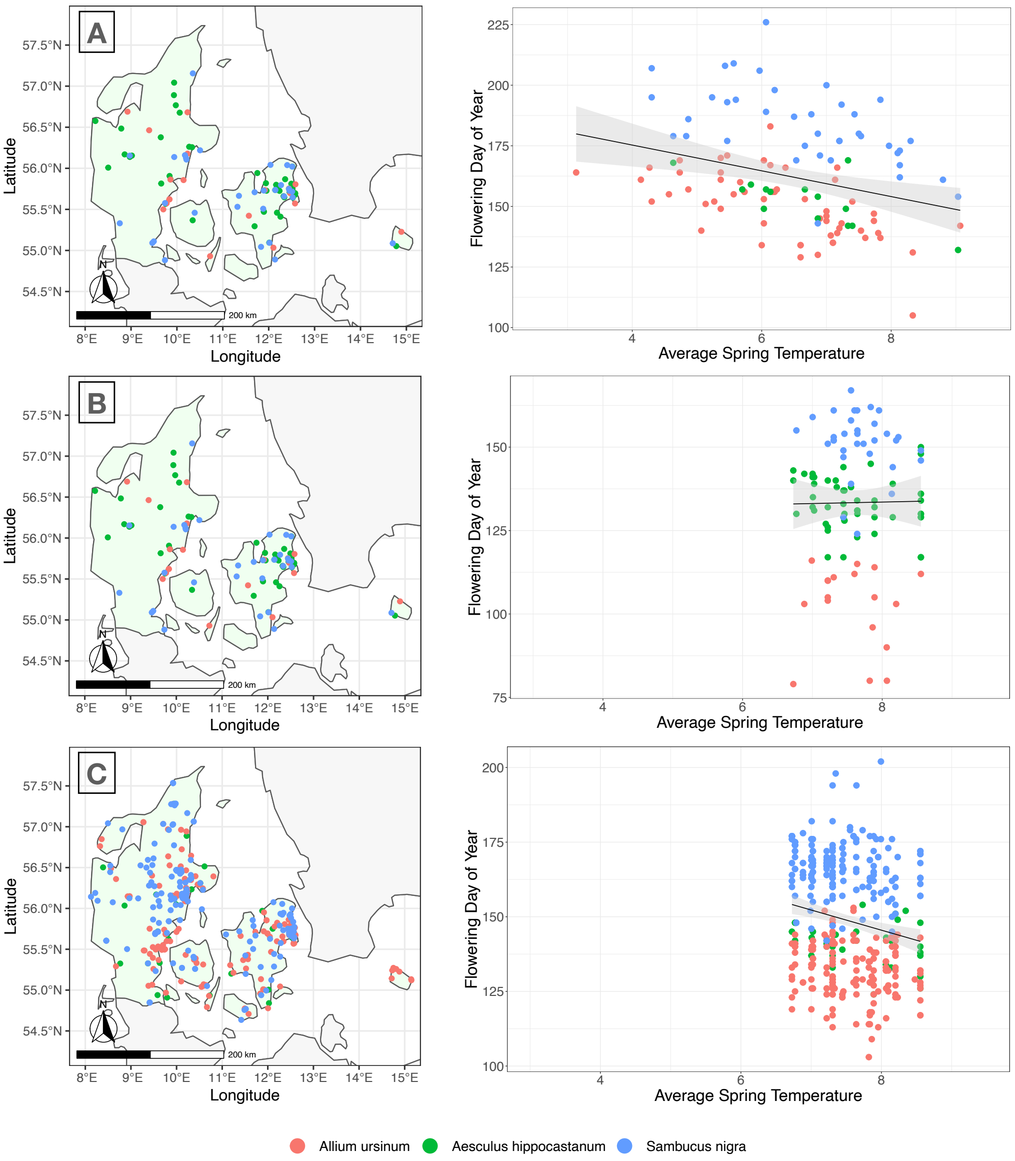

Fig. S2 Distribution of data points across Denmark as documented by (A) herbarium specimens; (B) citizen science observations of first flowering dates; and (C) photographs uploaded to iNaturalist. The herbarium data show the widest range of temperatures. The iNaturalist data set has the greatest number of observations. There are strong temperature effects on flowering times for the herbarium specimens and the iNaturalist photographs, but not for the citizen science observations

Online Resource 3

Case study – Phenology networks and resources for citizen science

Phenology networks exist around the world and contribute to advances in phenological research, such as understanding national and continental-scale patterns of phenological change and improving forecasts of phenology (Crimmins et al. 2020; Menzel et al. 2020; Gerst et al. 2020). These networks frequently facilitate citizen science as a part of their work. ClimateWatch (Australia), SeasonWatch (India), Naturekalender (Netherlands), Albertine Rift Program (Uganda), the Chinese Phenological Observation Network (CPON), Bhutan Phenology Network (Bhutan), Nature's Calendar (United Kingdom), and the USA National Phenology Network (United States) have collectively engaged tens of thousands of participants to contribute tens of millions of phenological observations around the world.

The USA National Phenology Network (Fig. S3) (Crimmins et al. 2022) specifically cultivates participation by and collaboration with pre-existing groups, such as students at schools or guides at public gardens, resulting in more frequent observations, and therefore more precise estimates of phenophases. This teamworks improve data quality, especially the identification of species and phenophases, often resulting in many years of observations (Crimmins et al. 2022).

Many citizen science programs allow participants to report observations on species of their choosing. Researchers and managers can also collaborate with phenology networks to initiate campaigns to collect data to address particular questions or applications. For example, a USA National Phenology Network campaign encouraging observations of forest trees facilitated comparisons between species-level ground-based observations and landscape-level observations from satellites (Elmore et al. 2016).

Researchers interested in adding citizen science elements to their own research can contact phenology networks and citizen science associations in their countries for help (Storksdieck et al. 2016). Many of these organizations have online resources, toolkits, trainings, and communities of practice (e.g., CitizenScience.org or usanpn.org).

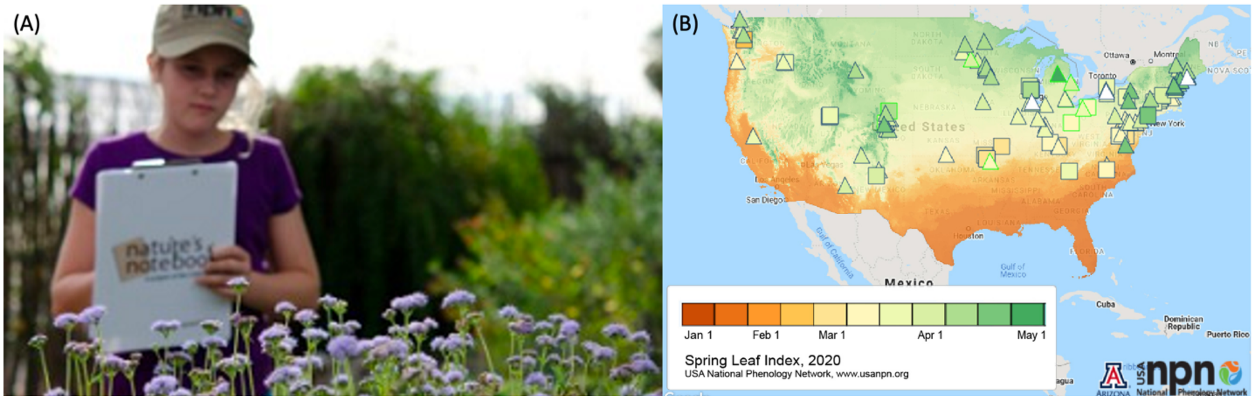

Fig. S3 (A) Nature's Notebook observer and (B) USA-NPN 2020 Spring Leaf Index map. Spring Leaf Index indicates the day of year that conditions associated with leaf-out in early-season plants were reached. Triangles represent observations of common lilac (*Syringa vulgaris*); squares represent observations of red rothomagensis lilac (*Syringa chinensis*; squares). Images courtesy of USA-NPN

Online Resource 4

Case study – Assessing phenology across spatial scales

Combining satellite and ground observations can help researchers investigate the influences of broad-scale atmospheric and local drivers of phenology on ecosystem processes, such as water, carbon, and nutrient cycling (Zeng et al. 2020; Peng et al. 2017; Elmore et al. 2016; Friedland et al. 2018). However, such combinations are difficult, given the inherent spatial and temporal differences between the two data types. Despite such differences, these data types can be integrated with sufficient care (Liang et al. 2011).

For example, Liang et al. collected ground observations in a Wisconsin forest for representative tree species over areas equal or greater in size than the remotely sensed pixels (Fig. S4). The team recorded observations at regular intervals and used a slightly modified version of the BBCH scale, which gave a continuous status-based scale of leaf development (Denny et al. 2014). They then blended ground data to produce landscape phenology "pixels" that were fully comparable in space and time with satellite pixel data and accurate to within two days (Denny et al. 2014; Liang et al. 2011).

Fixed cameras, such as employed in the PhenoCam network or video monitoring of diadromous fishes in coastal streams, can collect remote sensing-based phenological data at intermediate resolutions that further facilitate integration across land and stream-scapes and local scales (Brown et al. 2016; Klosterman et al. 2014; Dalton et al. 2022). Cameras can capture plant, fish or mammal phenology at resolutions similar to the scale of remotely-sensed pixels, but still allow researchers to distinguish among species. Cameras also capture vegetation color (most often greenness), and snow pack coverage similar to the measurements captured by satellite images (Zimova et al. 2020). Together these intermediate data can further help to bridge gaps between satellites and ground observations at multiple sites (Tian et al. 2021).

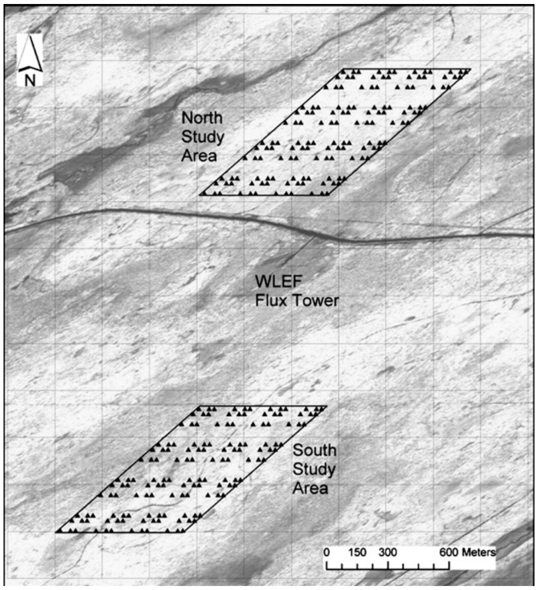

Fig. S4 Sampling design (in sinusoidal projection) to match ground-based phenological observations with MODIS pixel grids (250 × 250-m squares). The background image is 2.4-m resolution NDVI derived from a May 18, 2007 QuickBird image. Figure from Liang et al. (2011)

Online Resource 5

Case study – Tracking seasonal migrations of anadromous fishes

Anadromous fishes including salmon (*Oncorhynchus* sp. and *Salmo* sp.) and river herring (*Alosa pseudoharengus* and *A. aestivalis*) are valued as subsistence, recreational, and commercial fisheries by coastal communities around the world (Daigle et al. 2019; Hare et al. 2021; Miyakoshi et al. 2004; Bal et al. 2017). Anadromous fishes have complex migration patterns and life histories that span marine and freshwater environments and require aquatic connectivity and access to freshwater spawning habitat (Martins et al. 2012).

During spring in New England, USA, adult river herring migrate from the ocean into coastal streams and ponds to spawn. Juvenile fish emigrate back to sea in late summer and fall. Researchers monitor spring herring runs at fish ladders, dams, and other structures using electronic and video counters, and other methods (Fig. S5). In Massachusetts, USA, a network of town-appointed citizen scientists known as "herring wardens" measure stream temperature and visually track the number of fish on a daily basis (Rideout et al. 1979). These counts provide valuable records that span decades.

Recent studies have combined long-term monitoring data (1990-2017) from 12 Massachusetts sites collected by herring wardens, state biologists, and other organizations. Multivariate linear models, which included population size and counting method as fixed effects, assessed how herring run initiation, peak, end, and duration changed over time and in relation to oceanographic, climatic, hydrological, and lunar cycle factors (Legett et al. 2021; Dalton et al. 2022).

At most locations run timing did not change over time and was best predicted by winter severity—herring migrated later in springs following colder, longer winters. Earlier runs occurred at some sites where fish numbers increased after dam removals and improvements to aquatic connectivity. Studies at more northern (Huntington et al. 2003) and southern (Lombardo et al. 2020) herring sites along the U.S. Atlantic coast have detected stronger phenological advancements in spring migration. In contrast, data collected by recreational and commercial anglers in European rivers reveal later migration phenology in mature Atlantic salmon (*Salmo salar*) and declines in body condition and size (Bal et al. 2017; Todd et al. 2012).

Harvest levels, population restoration, barriers to passage, and difficulties distinguishing species can mask climate responses and complicate understanding of responses to climate change and other influences on their populations. Emerging technologies such as eDNA are advancing the field of fish migration phenology and helping researchers address these challenges. They can allow detections across a wider range of locations and time periods (Yamasaki et al. 2017; Thalinger et al. 2019; Maruyama et al. 2018) and help researchers to separate the phenology of closely related species (Nakagawa et al. 2018; Cilleros et al. 2019).

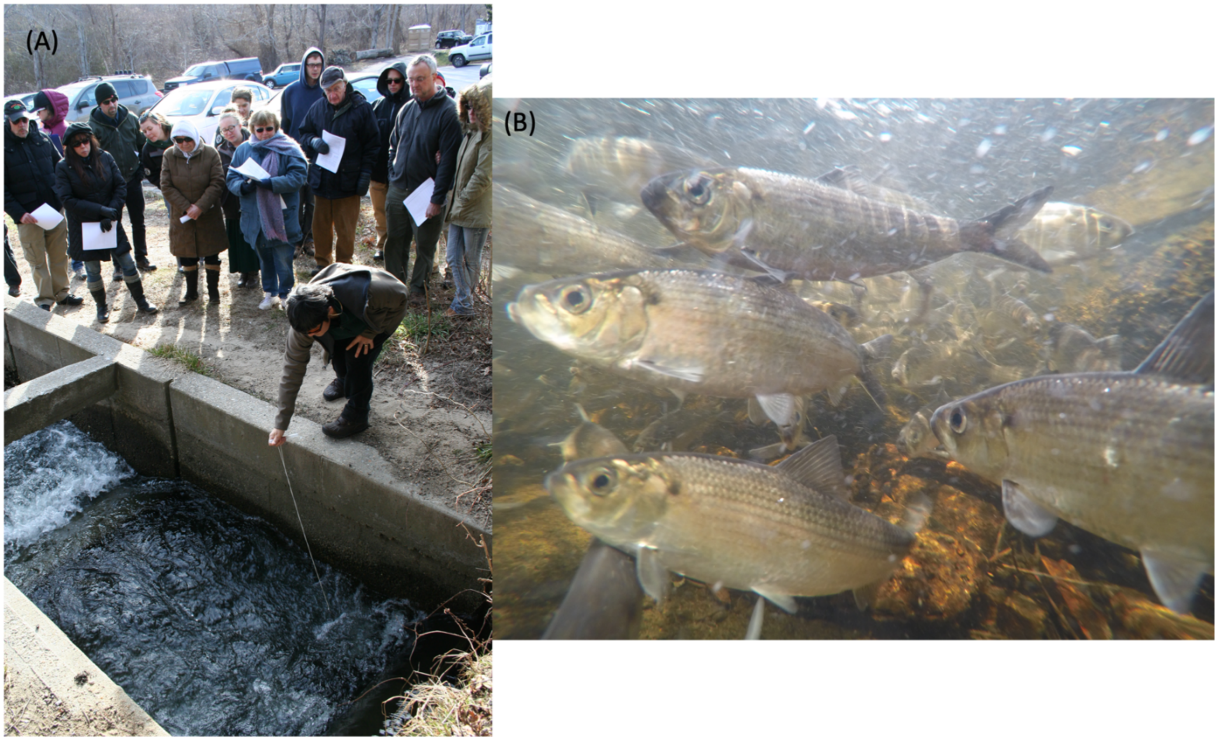

Fig. S5 (A) A training for “herring wardens” at a fish ladder on the Mashpee River in Mashpee, Massachusetts, USA and (B) adult alewife (*Alosa pseudoharengus*) schooling at Parker River, Massachusetts. Images courtesy of Jo Ann Muramoto (A) and Matthew Devine (B)

References

Bal G, Montorio L, Rivot E, Prévost E, Baglinière JL, Nevoux M (2017) Evidence for long-term change in length, mass and migration phenology of anadromous spawners in F rench A tlantic salmon Salmo salar. Journal of Fish Biology 90 (6):2375-2393

Brown TB, Hultine KR, Steltzer H, Denny EG, Denslow MW, Granados J, Henderson S, Moore D, Nagai S, SanClements M (2016) Using phenocams to monitor our changing Earth: toward a global phenocam network. Front Ecol Environ 14 (2):84-93

Cilleros K, Valentini A, Allard L, Dejean T, Etienne R, Grenouillet G, Iribar A, Taberlet P, Vigouroux R, Brosse S (2019) Unlocking biodiversity and conservation studies in high-diversity environments using environmental DNA (eDNA): A test with Guianese freshwater fishes. Molecular Ecology Resources 19 (1):27-46

Crimmins T, Denny E, Posthumus E, Rosemartin A, Croll R, Montano M, Panci H (2022) Science and management advancements made possible by the USA National Phenology Network's Nature's Notebook platform. Bioscience 72:908-920. doi:10.1093/biosci/biac061

Crimmins TM, Gerst KL, Huerta DG, Marsh RL, Posthumus EE, Rosemartin AH, Switzer J, Weltzin JF, Coop L, Dietschler N (2020) Short-term forecasts of insect phenology inform pest management. Annals of the Entomological Society of America 113 (2):139-148

Daigle JJ, Michelle N, Ranco DJ, Emery MR (2019) Traditional lifeways and storytelling: Tools for adaptation and resilience to ecosystem change. Human Ecology 47 (5):777-784

Dalton RM, Sheppard JJ, Finn JT, Jordaan A, Staudinger MD (2022) Phenological variation in spring migration timing of adult alewife in coastal Massachusetts. Marine and Coastal Fisheries 14:e10198

Denny EG, Gerst KL, Miller-Rushing AJ, Tierney GL, Crimmins TM, Enquist CA, Guertin P, Rosemartin AH, Schwartz MD, Thomas KA, Weltzin JF (2014) Standardized phenology monitoring methods to track plant and animal activity for science and resource management applications. Int J Biometeorol 58:591-601. doi:10.1007/s00484-014-0789-5

Ellwood ER, Gallinat AS, McDonough MacKenzie C, Miller T, Miller-Rushing AJ, Polgar C, Primack RB (2021) Plant and bird phenology and plant occurence from 1851 to 2020 (non-continuous) in Thoreau’s Concord, Massachusetts. Ecology In press

Ellwood ER, Primack RB, Talmadge ML (2010) Effects of climate change on spring arrival times of birds in Thoreau's Concord from 1851 to 2007. The Condor 112 (4):754-762. doi:10.1525/cond.2010.100006

Elmore AJ, Stylinski CD, Pradhan K (2016) Synergistic use of citizen science and remote sensing for continental-scale measurements of forest tree phenology. Remote Sensing 8 (6):502

Friedland KD, Mouw CB, Asch RG, Ferreira ASA, Henson S, Hyde KJ, Morse RE, Thomas AC, Brady DC (2018) Phenology and time series trends of the dominant seasonal phytoplankton bloom across global scales. Global Ecology and Biogeography 27:551-569

Gerst KL, Crimmins TM, Posthumus EE, Rosemartin AH, Schwartz MD (2020) How well do the spring indices predict phenological activity across plant species? Int J Biometeorol 64 (5):889-901

Hare JA, Borggaard DL, Alexander MA, Bailey MM, Bowden AA, Damon-Randall K, Didden JT, Hasselman DJ, Kerns T, McCrary R (2021) A review of river herring science in

support of species conservation and ecosystem restoration. Marine and Coastal Fisheries 13 (6):627-664

Heberling JM, McDonough MacKenzie C, Fridley JD, Kalisz S, Primack RB (2019) Phenological mismatch with trees reduces wildflower carbon budgets. Ecol Lett 22 (4):616-623

Henckel L, Bradter U, Jönsson M, Isaac NJ, Snäll T (2020) Assessing the usefulness of citizen science data for habitat suitability modelling: Opportunistic reporting versus sampling based on a systematic protocol. Diversity and Distributions 26 (10):1276-1290

Huntington TG, Hodgkins G, Dudley R (2003) Historical trend in river ice thickness and coherence in hydroclimatological trends in Maine. Climatic Change 61 (1):217-236

Iwanycki Ahlstrand N, Primack RB, Tøttrup AP (2022) A comparison of herbarium and citizen science phenology datasets for detecting response of flowering time to climate change in Denmark. Int J Biometeorol 66 (5):849-862

Klosterman S, Hufkens K, Gray J, Melaas E, Sonnentag O, Lavine I, Mitchell L, Norman R, Friedl M, Richardson A (2014) Evaluating remote sensing of deciduous forest phenology at multiple spatial scales using PhenoCam imagery. Biogeosciences 11 (16):4305-4320

Legett HD, Jordaan A, Roy AH, Sheppard JJ, Somos-Valenzuela M, Staudinger MD (2021) Daily patterns of river herring (*Alosa* spp.) spawning migrations: Environmental drivers and variation among coastal streams in Massachusetts. Transactions of the American Fisheries Society 150:501-513

Liang L, Schwartz MD, Fei S (2011) Validating satellite phenology through intensive ground observation and landscape scaling in a mixed seasonal forest. Remote Sens Environ 115 (1):143-157

Lombardo SM, Buckel JA, Hain EF, Griffith EH, White H (2020) Evidence for temperature-dependent shifts in spawning times of anadromous alewife (*Alosa pseudoharengus*) and blueback herring (*Alosa aestivalis*). Canadian Journal of Fisheries and Aquatic Sciences 77 (4):741-751

Martins EG, Hinch SG, Cooke SJ, Patterson DA (2012) Climate effects on growth, phenology, and survival of sockeye salmon (*Oncorhynchus nerka*): a synthesis of the current state of knowledge and future research directions. Reviews in Fish Biology and Fisheries 22 (4):887-914

Maruyama A, Sugatani K, Watanabe K, Yamanaka H, Imamura A (2018) Environmental DNA analysis as a non-invasive quantitative tool for reproductive migration of a threatened endemic fish in rivers. Ecology and evolution 8 (23):11964-11974

Menzel A, Yuan Y, Matiu M, Sparks T, Scheifinger H, Gehrig R, Estrella N (2020) Climate change fingerprints in recent European plant phenology. Glob Change Biol 26 (4):2599-2612

Miller-Rushing AJ, Inouye DW, Primack RB (2008) How well do first flowering dates measure plant responses to climate change? The effects of population size and sampling frequency. J Ecol 96:1289-1296

Miyakoshi Y, Koyama T, Aoyama T, Sakakibara S, Kitada S (2004) Estimates of numbers of masu salmon caught by recreational fishermen in the coastal area off Iburi, Hokkaido, Japan. Fisheries Science 70 (1):87-93

Nakagawa H, Yamamoto S, Sato Y, Sado T, Minamoto T, Miya M (2018) Comparing local-and regional-scale estimations of the diversity of stream fish using eDNA metabarcoding and conventional observation methods. Freshwater Biology 63 (6):569-580

Peng D, Zhang X, Wu C, Huang W, Gonsamo A, Huete AR, Didan K, Tan B, Liu X, Zhang B (2017) Intercomparison and evaluation of spring phenology products using National Phenology Network and AmeriFlux observations in the contiguous United States. Agricultural and Forest Meteorology 242:33-46
Primack RB, Miller-Rushing AJ (2012) Uncovering, collecting, and analyzing records to investigate the ecological impacts of climate change: A template from Thoreau's Concord. Bioscience 62:170-181
Rideout SG, Johnson JE, Cole CF (1979) Periodic counts for estimating the size of the spawning population of alewives, *Alosa pseudoharengus* (Wilson). Estuaries 2 (2):119-123
Soroye P, Ahmed N, Kerr JT (2018) Opportunistic citizen science data transform understanding of species distributions, phenology, and diversity gradients for global change research. Glob Change Biol 24 (11):5281-5291
Storksdieck M, Shirk JL, Cappadonna JL, Domroese M, Göbel C, Haklay M, Miller-Rushing AJ, Roetman P, Sbrocchi C, Vohland K (2016) Associations for citizen science: regional knowledge, global collaboration. Citizen Science: Theory and Practice 1 (2):10. doi:http://dx.doi.org/10.5334/cstp.55
Thalinger B, Wolf E, Traugott M, Wanzenböck J (2019) Monitoring spawning migrations of potamodromous fish species via eDNA. Scientific reports 9 (1):1-11
Tian F, Cai Z, Jin H, Hufkens K, Scheifinger H, Tagesson T, Smets B, Van Hoolst R, Bonte K, Ivits E (2021) Calibrating vegetation phenology from Sentinel-2 using eddy covariance, PhenoCam, and PEP725 networks across Europe. Remote Sens Environ 260:112456
Todd CD, Friedland KD, MacLean JC, Whyte BD, Russell IC, Lonergan ME, Morrissey MB (2012) Phenological and phenotypic changes in Atlantic salmon populations in response to a changing climate. ICES Journal of Marine Science 69 (9):1686-1698
Yamasaki E, Altermatt F, Cavender-Bares J, Schuman MC, Zuppinger-Dingley D, Garonna I, Schneider FD, Guillén-Escribà C, van Moorsel SJ, Hahl T (2017) Genomics meets remote sensing in global change studies: monitoring and predicting phenology, evolution and biodiversity. Current Opinion in Environmental Sustainability 29:177-186
Zeng L, Wardlow BD, Xiang D, Hu S, Li D (2020) A review of vegetation phenological metrics extraction using time-series, multispectral satellite data. Remote Sens Environ 237:111511
Zimova M, Sirén APK, Nowak JJ, Bryan AM, Ivan JS, Morelli TL, Suhrer SL, Whittington J, Mills LS (2020) Local climate determines vulnerability to camouflage mismatch in snowshoe hares. Global Ecology and Biogeography 29 (3):503-515. doi:https://doi.org/10.1111/geb.13049
